# Influence of Porosities of 3D Printed Titanium Implants on the Tensile Properties: Correspondence

**DOI:** 10.51894/001c.127958

**Published:** 2025-01-07

**Authors:** Hinpetch Daungsupawong, Viroj Wiwanitkit

**Affiliations:** 1 Private Academic Consultant Phonhong, Lao People’s Democratic Republic; 2 Department of Research Analytics Saveetha Dental College and Hospitals, Saveetha Institute of Medical and Technical Sciences, Saveetha University, Chennai, India

**Keywords:** titanium, porosity, tendon healing

Dear Editor,

This is a comment on a published research on “Influence of Porosities of 3D Printed Titanium Implants on the Tensile Properties in a Rat Tendon Repair Model.”^1^ The study on the effect of pore size on tendon repair fixation with 3D printed titanium implants gives useful information for biomaterial design. However, certain methodological difficulties must be addressed. First, while the sample size of 24 rats is appropriate for preliminary findings, it may lack statistical power to detect minor variations between groups, particularly in mechanical testing outcomes. To strengthen the study’s findings, future studies should consider increasing the sample size to improve statistical power and ensure that minor differences are detectable. Furthermore, using only one control group (no pore size) hinders our capacity to properly grasp the relative effectiveness of the implants, as differences in biological response may be caused by factors unrelated to pore size. It would be beneficial to include additional control groups, such as a group with small pore size, and a commercially available tendon repair fixation device to better compare the influence of different pore sizes on tendon healing and mechanical outcomes.

Furthermore, while mechanical testing and histological analysis yield relevant results, the study does not give detailed reporting of any confounding factors that could have influenced the results. Differences in surgical technique, individual animal variability, and postoperative care can all have an impact on healing and mechanical outcomes. To address this, future studies should consider standardizing the surgical procedure, clearly monitoring postoperative protocols, and tracking any animal-specific variables, such as weight and general health, that could affect healing outcomes. Furthermore, while the 12-week period is appropriate for initial evaluation, it may be insufficient to accomplish long-term tendon integration and functional recovery, both of which are required for putting these findings into clinical practice. Longer follow-up periods, such as 24 weeks or more, would provide a more comprehensive understanding of tendon integration and the mechanical durability of the implants over time.

Important questions for broader discussion: How do 3D printed titanium implants differ from typical tendon repair fixation procedures in terms of mechanical properties? How does pore size affect cell activity and integration with host tissue? Furthermore, how may results on pore size influence the future design of biomaterials for tendon healing and other regenerative medicine applications? Previous studies have shown that larger pore size can enhance cellular infiltration and tissue integration, while smaller pores may limit vascularization and tissue ingrowth, ultimately affecting healing conditions.^2,3^

Future study should focus on expanding sample sizes and integrating several control groups to have a more detailed knowledge of how pore size affects tendon recovery. Additionally, alternative metrics for measuring mechanical strength, such as fatigue testing, stiffness measurements, and load-to-failure testing, should be included to provide a more thorough evaluation of implant performance under physiologically relevant conditions. Longitudinal investigations conducted after 12 weeks of recovery will provide information about the long-term performance and integration of implants. Studying the biological mechanisms that encourage tissue growth in response to varying hole sizes may lead to the development of novel biomaterials that improve tendon healing outcomes. Exploring hybrid materials or coatings that combine the mechanical advantages of titanium with bioactive chemicals could lead to more cell proliferation and tissue regeneration, ultimately improving the efficacy of tendon restoration procedures.

## Authors’ contribution

HD 50 % ideas, writing, analyzing, approval

VW 50 % ideas, supervision, approval

## Conflict of interest

Authors declare no conflict of interest

## AI declaration

The authors use AI for language editing and translation of the article.
